# Lateral Surgical Approach to Lumbar Intervertebral Discs in an Ovine Model

**DOI:** 10.1100/2012/873726

**Published:** 2012-09-17

**Authors:** David Oehme, Tony Goldschlager, Jeffrey Rosenfeld, Andrew Danks, Peter Ghosh, Anne Gibbon, Graham Jenkin

**Affiliations:** ^1^The Ritchie Centre, Monash Institute of Medical Research (MIMR), Monash University, Clayton, VIC 3168, Australia; ^2^Department of Neurosurgery, Monash Medical Centre, Clayton, VIC 3168, Australia; ^3^Department of Neurosurgery, The Alfred Hospital, Prahran, VIC 3181, Australia; ^4^Proteobioactives Research Laboratories, Brookvale, NSW 2100, Australia; ^5^Monash Animal Services, Monash University, Clayton, VIC 3168, Australia

## Abstract

The sheep is becoming increasingly used as a large animal model for preclinical spine surgery studies. Access to the ovine lumbar intervertebral discs has traditionally been via an anterior or anterolateral approach, which requires larger wound incisions and, at times, significant abdominal retraction. We present a new minimally invasive operative technique for a far-lateral approach to the ovine lumbar spine that allows for smaller incisions, excellent visualisation of intervertebral discs, and minimal abdominal retraction and is well tolerated by animals with minimal morbidity.

## 1. Introduction

The sheep spine shares many similarities, both anatomically and biochemically, to the human spine, making it increasingly popular as a large animal model for preclinical spine surgery studies [1–3]. The ovine spine has been used as a model for disc degeneration [4–9], to test novel implant devices [10–12], and as a preclinical model for biological therapies such as stem cell treatments or administration of growth factors [13–16]. 

Posterior approaches to the lumbar intervertebral discs, commonly used in human surgery, are difficult in the sheep due to the presence of the spinal cord within the lumbar spinal canal and ossification of the posterior longitudinal ligament (PLL). For this reason, the ovine lumbar intervertebral discs have traditionally been accessed via an anterior or anterolateral approach [5, 17]. This retroperitoneal or transperitoneal approach carries risks that include bowel and great vessel injury, neural injury, and hernia formation. The procedure also requires a large abdominal incision with greater retraction of abdominal viscera, which can be harmful to the animal. 

We describe here a new minimally invasive lateral approach to the sheep lumbar spine, which affords easy access to the lumbar intervertebral discs and is well tolerated by the animals. The technique allows for a small focused incision, which is away from dependent abdominal areas, decreasing the risk of postoperative hernia and abdominal and wound complications. A similar minimally invasive extreme lateral approach has gained popularity in humans, using a transmuscular (transpsoas) route with neuromonitoring guidance [18–20]. However, in the ovine lateral approach described herein, the psoas muscle can be easily retracted without the requirement for neural monitoring. This new surgical technique provides an alternative to traditional anterior and anterolateral approaches to the sheep lumbar spine. 

## 2. Methods

This procedure has been undertaken in 95 two-year-old East Friesian/Merino Cross wethers (weight range 55–90 kilograms) to perform lumbar annular disc injury in order to elicit disc degeneration (*n* = 86), perform discectomy procedures (*n* = 9), and implant stem cells for novel therapies (*n* = 86). In 72 of these animals, the procedure has been performed bilaterally from the left side to illicit disc degeneration, and then three months later from the contralateral right side to inject regenerative stem cells. This approach has allowed access from L1 to L6. In the course of our experiments, animals are typically monitored for at least six months following surgery, prior to postmortem. 

## 3. Surgical Anatomy

**Figure 1:**
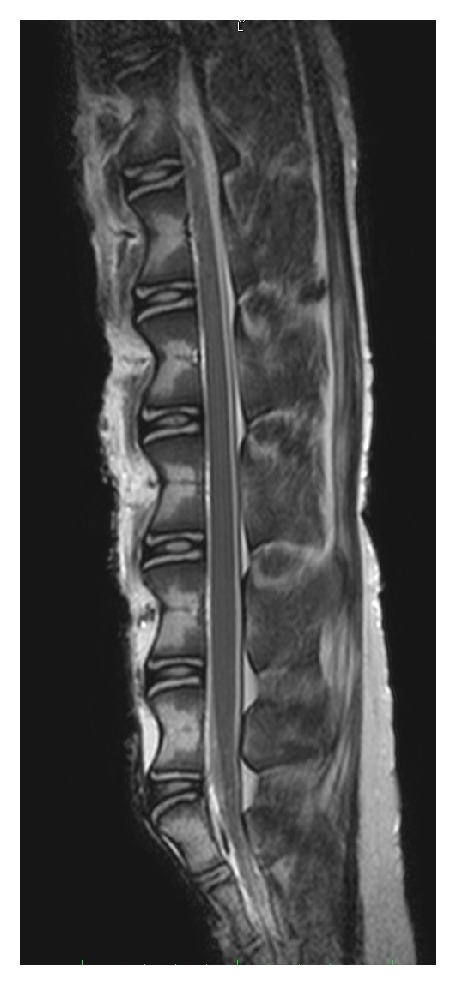
3T sagittal T2-weighted MRI of ovine lumbar spine demonstrating concave elongated vertebral bodies, intervertebral discs, and the persistence of the spinal cord into the sacral region.

The sheep characteristically has six lumbar vertebrae although seven may be apparent with the presence of transitional lumbosacral anatomy. Vertebral body to intervertebral disc height ratio in adult sheep is greater than that in humans vertebral body heights commonly exceed 40 mm (mean 42.49, SD 2.36) whilst disc heights are usually only 4-5 mm (mean 4.48, SD 0.66). The discs and endplates appear as bulbous convex expansions in between concave elongated vertebral bodies (Figure 1). The sheep transverse processes are larger than in the human, are easily palpable, and are visible in the flank region, serving as useful landmarks when performing surgery. 

**Figure 2:**
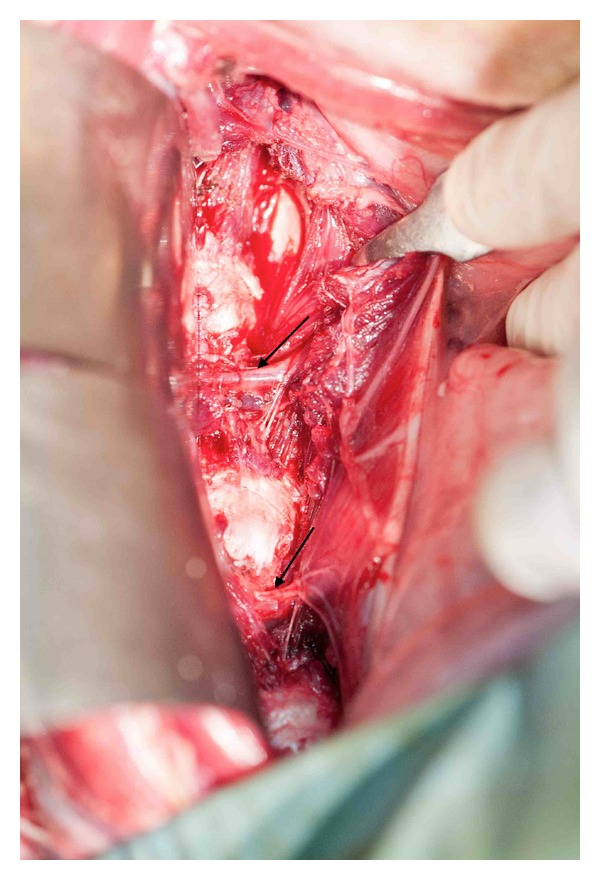
Intraoperative photo demonstrating radicular vessels at two levels (arrows) running horizontally across vertebral bodies 1 cm below intervertebral discs.

Radicular veins and arteries can be found running approximately 1 cm below the inferior endplates across the vertebral bodies and are variable in size and number (Figure 2). When torn, bleeding can be profuse but is controlled with bipolar diathermy. Muscular insertions into the lower lumbar vertebral bodies are usually thick and tendinous, whilst those higher in the lumbar spine are thin and easily divided. This lateral surgical approach can be performed, without limitation, from the right or left sides. 

The spinal cord continues into the sacral region in the sheep, and one must be aware of this when passing instruments through the disc space so as not to cause cord injury. From our observations, the PLL is often ossified, or at least partially calcified, and serves as a protective barrier in this situation.

## 4. Surgical Technique

### 4.1. Preparation

All surgical and experimental procedures were approved by the Monash Medical Centre Animal Ethics Committee as conforming to the Australian code of practice for the care and use of animals for scientific purposes 7th Edition, 2004. 

Sheep are fasted for 24 hours in order to prevent abdominal distension and aspiration of rumen fluids during surgery. Animals are sedated with intravenous medetomidine hydrochloride (Domitor—0.015–0.02 mg/kg), to facilitate transport to the operating theatre, followed by intravenous injection of thiopentone (10–13 mg/kg) for anaesthetic induction. An endotracheal tube is inserted and anaesthesia maintained by isofluorane (2-3% in oxygen) inhalation. All animals receive perioperative intravenous antibiotic (amoxicillin 1 g IV). We do not use muscle relaxation. 

Once anaesthetised, the sheep is placed on the operating table in the lateral position. The lateral abdomen (flank) and spine is shaved and prepared with chlorhexidine and alcoholic-iodide antiseptic wash followed by sterile draping. Local anaesthetic (bupivicaine 0.5%) is subcutaneously injected around the incision site. Strict sterile precautions are maintained at all times.

### 4.2. Lateral Approach to the Lumbar Intervertebral Discs

**Figure 3:**
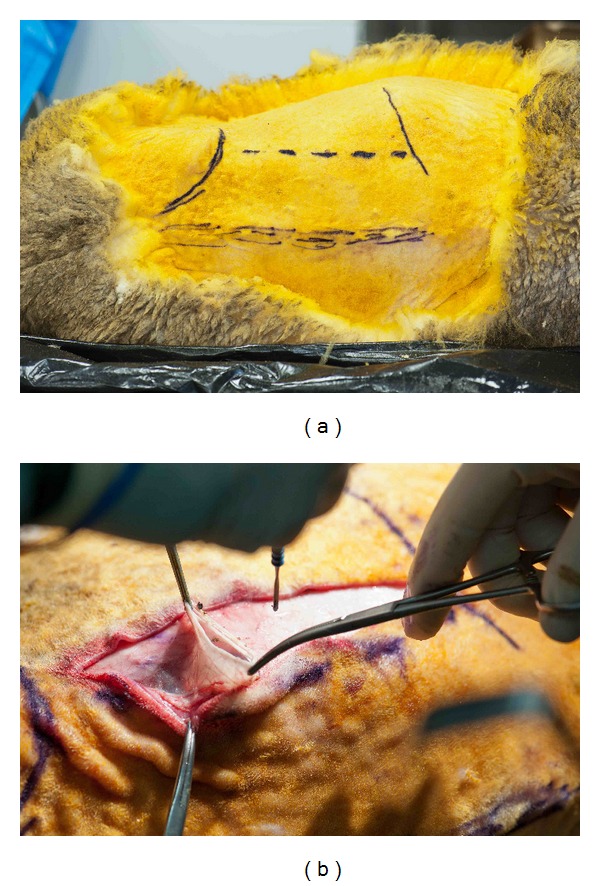
(a) Preoperative photo of sheep in right lateral position demonstrating lumbar spinous processes (lower dashed line), left lumbar transverse processes (upper dashed line), iliac crest (left), and costal margin (right). (b) Longitudinal incision made parallel to and 1 cm anterior to transverse processes. Lateral aspect of abdominal wall fascia and musculature partially opened.

Landmarks used for the incision are easily palpable; these are the iliac crest, lumbar transverse processes and the costo-vertebral angle (Figure 3(a)). A longitudinal incision parallel and 1cm anterior to the transverse processes is made (Figure 3(b)). The length and exact location of the incision is guided by the desired disc level to be reached. A 10cm incision will facilitate access to 3-4 levels, with incisions extending to the iliac crest facilitating access to the lower lumber spine, whilst those extending to the costo-vertebral angle allow access to the upper lumbar and lower thoracic spines. Disc levels from T12/L1 to L5/L6 can be accessed using this approach. Smaller focused incisions can be used to access single-disc levels. 

**Figure 4:**
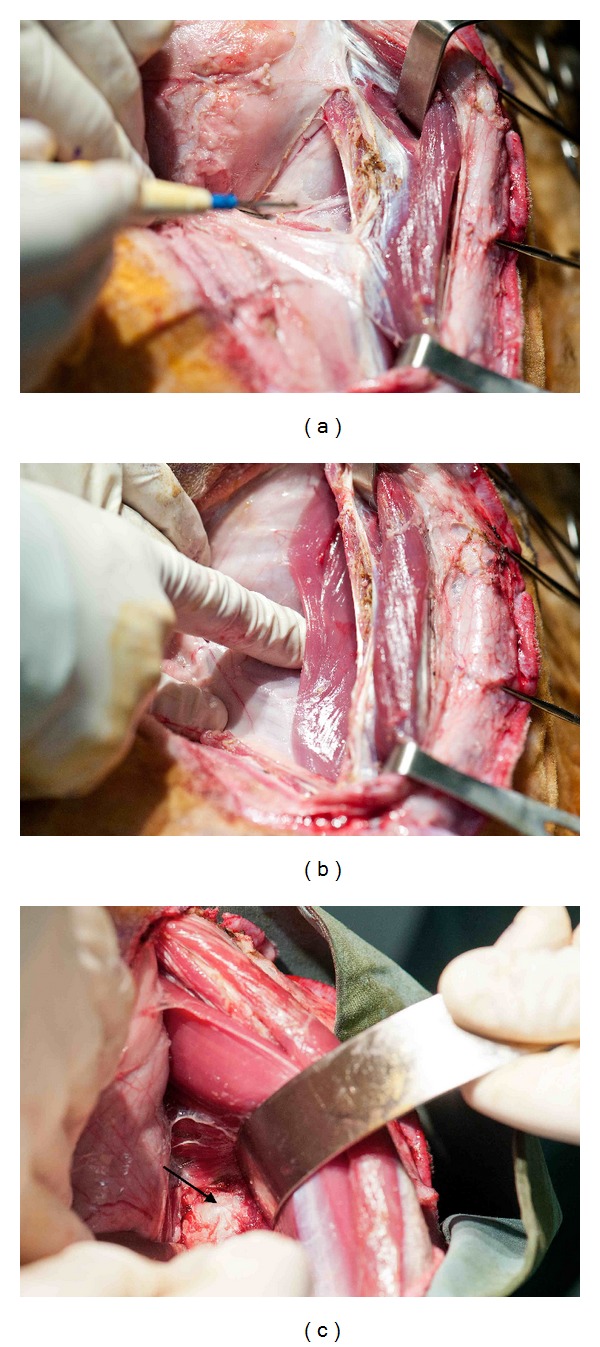
(a) Division of thoracolumbar fascia at its attachment to the transverse processes allows entry into retroperitoneal space. Erector spinae muscle is retracted laterally. (b) With the thoracolumbar fascia divided the psoas muscle becomes visible. The peritoneum can be carefully separated from the posterior abdominal wall musculature with blunt dissection. (c) Psoas muscle retracted laterally and abdomen retracted medially revealing the intervertebral disc (arrow).

Following sharp incision, the subcutaneous tissue is divided using monopolar diathermy. The lateral aspect of the abdominal wall musculature is also divided and, here, represents only a fatty fascial layer. The key to entering the retroperitoneal space, with minimal disruption to the peritoneum and its contents, is to longitudinally divide the thoracolumbar fascia at its attachment to the transverse processes (Figure 4(a)). With the thoracolumbar fascia divided, the quadratus lumborum and psoas muscles become visible. Traversing neurovascular bundles supplying the abdominal wall may be seen and attempts should be made to preserve these, although division does not appear to cause adverse effects. Now digital blunt dissection facilitates easy separation of the peritoneum from the posterior abdominal wall musculature and allows identification of the anterolateral aspect of the vertebral bodies and intervertebral discs (Figure 4(b)). 

We do not routinely suture small breaches in the fragile peritoneum or attempt to visualise the aorta and inferior vena cava, which can be palpated medially. As suggested by Baramki et al. [17], a Hohmann retractor with the tip positioned on the contralateral vertebral body is the best instrument to retract the abdominal contents and great vessels. The psoas and quadratus lumborum muscles are easily retracted posterolaterally, by the assistant, using a Diva retractor, further exposing the intervertebral discs (Figure 4(c)). 

The intervertebral bodies appear a long concave depressions, whilst the intervening intervertebral discs are convex swellings, which are easily palpated. By positioning retractors immediately over the discs, disruption of lumbar segmental arteries and veins, which are located approximately 1 cm caudal to the inferior endplate, can be avoided. If bleeding from these segmental vessels occurs, bipolar cautery affords simple haemostasis. Surgical loupe magnification with headlight illumination facilitates identification and preservation of exiting vessels and nerves.

At this stage, the exact disc level can be confirmed with lateral X-ray. Using the far-lateral approach described, the entire ipsilateral half of the intervertebral discs can be identified. Rotating the operating table away from the surgeon will open the view to the anterior portion of the disc. From our experience, muscular attachments over the disc can be easily swept aside using blunt dissection for levels L3/4 and above. For levels L4/5 and below, thicker tendinous muscular attachments require sharp division, using bipolar diathermy and scissors in order to expose the disc. Disc levels from T12/L1 to L5/6 can be easily reached using this lateral approach. The L6/S1 disc is difficult to access due to obstruction by the iliac crest. 

**Figure 5:**
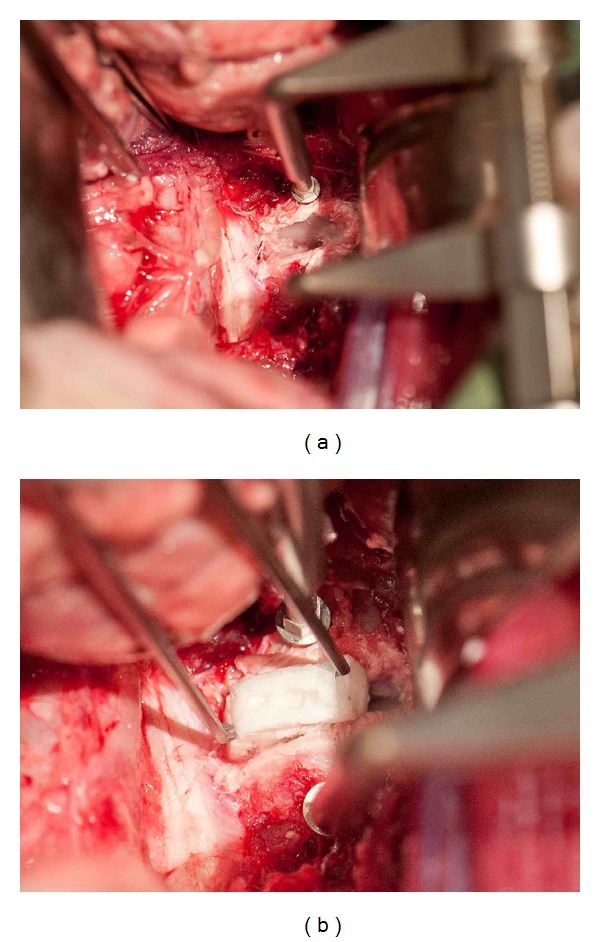
(a) Intraoperative photograph demonstrating intervertebral space following total discectomy. Distraction pins have been inserted into adjacent vertebral bodies to facilitate discectomy procedure. (b) Insertion of a mesenchymal progenitor cell-seeded polycaprolactone biological disc into the intervertebral space via lateral approach.

Once the desired procedure has been performed on the disc (s) (Figures 5(a) and 5(b)), and haemostasis is achieved, the wound is irrigated with Ringers' solution and a layered closure undertaken, using 2–0 Vicryl to the lateral abdominal wall tissues and continuous subcutaneous suture to the skin. Minimal blood loss occurs throughout this procedure, which, in our hands, is estimated to be approximately 10ml per animal. The entire procedure can be performed in less than one hour with minimal postoperative discomfort to the animal. 

### 4.3. Postoperative Management

We routinely use a transdermal fentanyl patch (Durogesic 75 mcg/hr) positioned in the inguinal region for postoperative analgesia. Further analgesia using buprenorphine (300 mcg IV) can be administered, but is seldom required. As soon as each sheep breathes spontaneously, following cessation of isofluorane anaesthesia, it is extubated and then transferred to a holding cage where it is given food when fully alert and standing. Medetomidine hydrochloride is reversed with atipamezole (Antisedan 0.06 mg/kg–0.08 mg/kg). The sheep is observed for approximately one hour following surgery, after which it can be returned to its holding pen with other animals. No significant problems with postoperative mobility or pain have been encountered; even when complete disc removal procedures are performed. 

## 5. Discussion

Lateral approaches to the human lumbar spine, such as the XLIF (extreme lateral lnterbody fusion) procedure, have gained popularity as a minimally invasive approach to the lumbar intervertebral discs. In the human lateral approach, a retroperitoneal transpsoas route is employed and requires real-time neuromonitoring to ensure safe passage through the psoas without damage to the lumbar plexus [19]. Postoperative motor nerve injury related to the approach is reported at 0.7 to 3.4%, whilst sensory symptoms occur in 1.6 to 10.3% [19, 21–23]. Many of these neural deficits are transient, however, neural injury remains a concern with this procedure. 

We have successfully performed the lateral approach described above in the sheep lumbar spine without complication in 95 animals, totalling 175 lumbar surgeries, as part of studies investigating stem cell mediated disc regeneration. To our knowledge, neural complications related to lumbosacral plexus injury have not occurred. Unlike in the human approach, which is transpsoas and requires neuromonitoring, the sheep psoas can be retracted without the requirement for neuromonitoring, with no apparent harm to the animals. 

We have not encountered any postoperative infections. We believe this is a result of extensive skin preparation prior to surgery with wide wool clipping and multiple antiseptic washes using chlorhexidine and alcoholic iodine. Blood loss has been minimal and there have been no anaesthetic complications or postoperative pain management concerns with this technique. We have not experienced any clinically apparent neural or great vessel injury, and postoperative hernia or wound dehiscence has not occurred to date. Our technique of not repairing peritoneal breaches has not led to clinically apparent bowel herniation or obstruction. 

## 6. Conclusions

This minimally invasive lateral approach to the anterior sheep lumbar spine affords easy access to the intervertebral discs from L1 to L6 and can be performed safely without significant morbidity to the animals. The procedure allows for good visualisation and surgical access to the intervertebral discs. This procedure provides an alternative to anterior approaches, which require larger incisions and greater abdominal retraction.
